# Polymorphic Microsatellite Markers for the Tetrapolar Anther-Smut Fungus *Microbotryum saponariae* Based on Genome Sequencing

**DOI:** 10.1371/journal.pone.0165656

**Published:** 2016-11-10

**Authors:** Taiadjana M. Fortuna, Alodie Snirc, Hélène Badouin, Jérome Gouzy, Sophie Siguenza, Diane Esquerre, Stéphanie Le Prieur, Jacqui A. Shykoff, Tatiana Giraud

**Affiliations:** 1 Laboratoire d’Ecologie Systématique Evolution, Univ. Paris-Sud, CNRS, AgroParisTech, Université Paris-Saclay, 91400, Orsay, France; 2 INRA, Laboratoire des Interactions Plantes-Microorganismes (LIPM), UMR441, Castanet-Tolosan, F-31326, France; 3 CNRS, Laboratoire des Interactions Plantes-Microorganismes (LIPM), UMR2594, Castanet-Tolosan, F-31326, France; 4 GenPhySE, Université de Toulouse, INRA, INPT, ENVT, Castanet-Tolosan, F-31326, France; University of Nebraska-Lincoln, UNITED STATES

## Abstract

**Background:**

Anther-smut fungi belonging to the genus *Microbotryum* sterilize their host plants by aborting ovaries and replacing pollen by fungal spores. Sibling *Microbotryum* species are highly specialized on their host plants and they have been widely used as models for studies of ecology and evolution of plant pathogenic fungi. However, most studies have focused, so far, on *M*. *lychnidis-dioicae* that parasitizes the white campion *Silene latifolia*. *Microbotryum saponariae*, parasitizing mainly *Saponaria officinalis*, is an interesting anther-smut fungus, since it belongs to a tetrapolar lineage (*i*.*e*., with two independently segregating mating-type loci), while most of the anther-smut *Microbotryum* fungi are bipolar *(i*.*e*., with a single mating-type locus). *Saponaria officinalis* is a widespread long-lived perennial plant species with multiple flowering stems, which makes its anther-smut pathogen a good model for studying phylogeography and within-host multiple infections.

**Principal Findings:**

Here, based on a generated genome sequence of *M*. *saponariae* we developed 6 multiplexes with a total of 22 polymorphic microsatellite markers using an inexpensive and efficient method. We scored these markers in fungal individuals collected from 97 populations across Europe, and found that the number of their alleles ranged from 2 to 11, and their expected heterozygosity from 0.01 to 0.58. Cross-species amplification was examined using nine other *Microbotryum* species parasitizing hosts belonging to *Silene*, *Dianthus* and *Knautia* genera. All loci were successfully amplified in at least two other *Microbotryum* species.

**Significance:**

These newly developed markers will provide insights into the population genetic structure and the occurrence of within-host multiple infections of *M*. *saponariae*. In addition, the draft genome of *M*. *saponariae*, as well as one of the described markers will be useful resources for studying the evolution of the breeding systems in the genus *Microbotryum* and the evolution of specialization onto different plant species.

## Introduction

Anther-smut fungi belonging to the genus *Microbotryum* sterilize their host plant by aborting ovaries and replacing pollen by fungal spores. Sibling *Microbotryum* species are highly specialized on their respective host plants and show strong post-mating reproductive isolation [1–3]. *Microbotryum* fungi have become model species in ecology and evolution of plant pathogenic fungi [4,5]. Although anther-smut disease occurs in hundreds of plant species in the Caryophyllaceae [6] and at least nine other plant families [7,8], most studies to date have focused on *M*. *lychnidis-dioicae*, which parasitizes the white campion *Silene latifolia*. This is the first fungal species in which distinct mating types were described [9], it has been used in classical genetic analyses for decades (*e*.*g*., [10–12]), and more recently as a model in disease transmission and metapopulation dynamics (*e*.*g*., [13,14]).

More recently, population genetics studies using microsatellite markers allowed elucidation of the genetic population structure of *M*. *lychnidis-dioicae* in Europe [15–17], patterns of post-glaciation expansion [18], and the invasion history of *M*. *lychnidis-dioicae* in North America [5,19]. The use of microsatellite markers also revealed a high prevalence of multiple infections by different fungal genotypes, which segregated in different stems of the same *Si*. *latifolia* host [20]. The co-infecting genotypes also revealed a high level of relatedness, suggesting competitive exclusion between unrelated fungal genotypes [21–23]. In addition, a reference genome of *M*. *lychnidis-dioicae* is now available [24,25], which provides opportunities for examining genomic changes within the genus. In particular, in this species, the two independent mating-type loci typical of basidiomycetes are trapped within a very large region of suppressed recombination on what have thus become the sex chromosomes, spanning ca. 90% of the chromosome length. These genomic data are consistent with previous findings based on marker segregation analyses and optical maps [26–28]. This large non-recombining region explains why *M*. *lychnidis-dioicae* is bipolar, with a single mating-type locus. Bipolarity is the rule for most *Microbotryum* species studied so far, suggesting similar linkage between mating-type loci [29].

Many resources are therefore available for the species *M*. *lychnidis-dioicae*, while the other anther-smut fungi remain poorly studied. Other *Microbotryum* species, however, deserve further investigations [7,30], and in particular *M*. *saponariae*, parasitizing *Saponaria ocymoides* and *Sa*. *officinalis* [2,8]. The common soapwort *Sa*. *officinalis*, with a broad distribution in Europe, is a large long-lived perennial plant with multiple stems, which makes it a particularly interesting subject for the study of multiple infections (*i*.*e*., multiple pathogen genotypes coexisting within a host) and phylogeography. In addition, *M*. *saponariae* has been recently identified as one of the few tetrapolar species (*i*.*e*., maintaining the independence of the two mating-type loci), recently identified, within the anther-smut *Microbotryum* fungi [29]. Of the available microsatellite markers for various *Microbotryum* fungi, very few cross-amplify on *M*. *saponariae* [15,17,31], and only four were polymorphic. Therefore, our aim here was to develop polymorphic microsatellite markers by generating a draft genome of *M*. *saponariae* using mate-pair Illumina sequencing. A similar approach to develop SNP markers has been used in other plant pathogenic fungi for studying multiple infections [32].

Microsatellite loci have been widely used as genetic markers due to their ubiquity, reproducibility, neutrality and high level of polymorphism [33]. These features make them valuable molecular markers for inference of evolutionary and demographic parameters [34]. However, for each new species, microsatellites often need to be isolated *de novo* and the characterization process of each locus can be time consuming and expensive given the need for numerous, locus-specific fluorescent primers. Recent methods using more simple and inexpensive approaches allow the isolation of polymorphic markers for large-scale multiplex assays. Here we isolated microsatellite markers using a high resolution and rapid approach with generic vector-specific fluorescent primers and unlabelled locus-specific primers [35], which makes the costs considerably lower.

The isolated microsatellite markers will allow studies for unravelling patterns of infection (*i*.*e*. single or multiple genotypes) within host, population structure and phylogeography of *M*. *saponariae*. In addition, the molecular and genomic resources obtained here will contribute to increasing our knowledge on the evolution of reproductive systems and host specialization of anther-smut fungi.

## Material and Methods

### Genome Sequencing

The *Microbotryum saponariae* genome sequenced in this study was originated from a fungal individual (strain 1053) collected on a host plant *Sa*. *officinalis* growing on the University Paris-Sud campus (Orsay, France) in 2012 and is available upon request. Diploid teliospores produced in the anther of a diseased plant were collected and cultivated on a nutrient-rich medium, as Potato Dextrose Agar (PDA) at 23°C for a few days. On nutritive media, fungal teliospores germinate and undergoes meiosis, each producing four haploid products, called sporidia, that replicate clonally. The sporidia harvested from PDA plates represent therefore the haploid products of many independent meiosis events of a single diploid fungal genotype. After adequate growth on PDA, sporidia were collected and stored in silica gel at -20°C until use. Fungal DNA was extracted using a hydraulic press (Carver, Inc., Wabash, USA) to lyse the cell walls and the Genomic-Tip 100/G kit (Qiagen^®^, CA, USA) following the manufacturer’s protocol. DNA purity was assessed by measuring 260/280 and 260/230 absorbance ratios with a NanoDrop^®^ spectrophotometer (2000; Thermo Scientific, Wilmington, USA), and DNA concentration was measured using a Qubit^®^ fluorometer (2.0; Thermo Scientific). Preparation of DNA libraries was performed by the GeT-Place INRA platform, Toulouse, France. Mate-pair libraries with insert sizes of 3kb were prepared with Illumina Nextera^TM^ Mate Pair Sample Prep kits, and sequencing was performed on a HiSeq 2000 Illumina^TM^ sequencer at a depth of coverage of 100X on average. The genome sequence of *M*. *saponariae* is available in GenBank under the accession number PRJEB11435.

### Detection of Microsatellite Loci in the Genome and Primer Design

Simple repeats were detected with RepeatMasker (v4.0.5; [36]) on the assembled genome. Only perfect di- and tri-nucleotide repeats of 8 to 12 repetitions were considered. Flanking regions of 200 bp were selected on each side of the nucleotide repeat. Thus, repeats located less than 200 bp from the contig extremity were discarded. Primers were designed using Primer3 [37] on 100 to 210 bp long fragments containing the target microsatellite sequences (S1 Table). For each microsatellite locus, up to five primer pairs were mapped to the target genome with GMAP (version 2014-10-16; [38]) using default parameters, and only pairs mapping to a single locus were further considered.

### Polymorphism Screening of Microsatellite Markers

**Fig 1:**
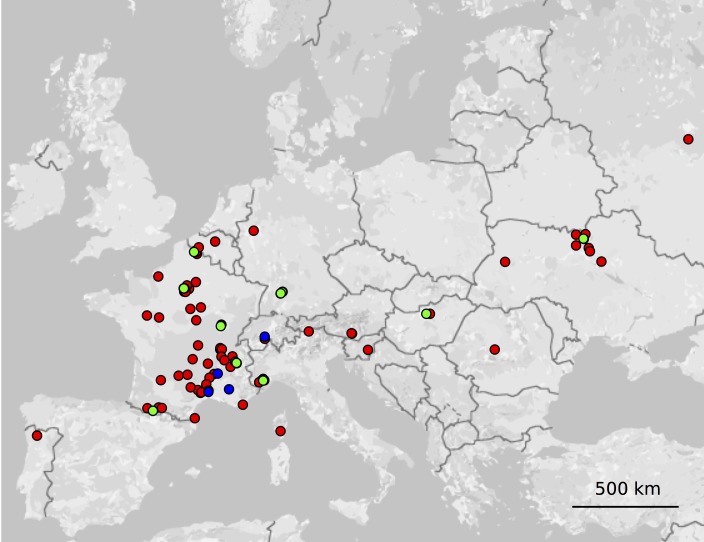
Map of the geographical location of *Microbotryum saponariae* individuals sampled in 97 populations of the host plants: 1) *Saponaria officinalis* (red circles, n = 92), including those used for the first polymorphism screening (green circles, n = 11), and 2) *Saponaria ocymoides* (blue circles, n = 5) in Europe. Fungal DNA was extracted from a sporulated anther of a diseased plant and was used to test the polymorphism of the 22 microsatellite markers.

For a first screening of polymorphism, a sample subset of eleven *M*. *saponariae* individuals were selected from different geographical locations across Europe (Fig 1, S2 Table), and DNA was extracted with the NucleoSpin® Soil kit (Macherey-Nagel, Germany). To detect polymorphic microsatellite markers, we used a simple and inexpensive approach that avoids generating fluorescent labels for all tested markers [35]. The eleven DNA samples, each at 5 ng.μL^-1^, were pooled as a template DNA for downstream applications. PCR amplifications were performed for each microsatellite marker with unlabelled specific primers using a high fidelity Taq DNA Polymerase Taq Pfu® (Promega, USA). We used a PCR touchdown program with 94°C for 5 min, 35 cycles of 94°C for 30 s, 62°C for 30°C with a decrease of 1°C at each cycle during 12 cycles and 50°C during 23 cycles, 72°C for 45 s, followed by 72°C for 5 min. Successful PCR amplifications were confirmed by 2% (w/v) agarose gel electrophoresis. PCR products for multiple individual microsatellite loci, amplified from the same DNA pool, were combined and purified using NucleoSpin® 96 PCR Clean-up (Macherey-Nagel, Germany), before ligation into pDrive vector (Qiagen^®^, USA). Ligation products were diluted 1/10 with deionised water and amplified again for each marker using the specific ‘reverse’ microsatellite primer and a universal fluorescently labelled primer, M13 forward (-40; 5’ GTTTTCCCAGTCACGAC 3’), targeting the plasmid insert flanking region. We used the same PCR touchdown program as described above. Labelled amplicons were diluted 1/225 and fractionated by capillary electrophoresis on an ABI PRISM X3730XL at the GENTYANE INRA platform, Clermont, France. Alleles of each microsatellite locus were identified and their size scored using GENEMAPPER 5.0 (Applied Biosystems^TM^, Foster city, USA).

### PCR Multiplex Assembling

For the assignment of PCR multiplexes we defined four amplification size ranges: 1) 100 to 115 bp; 2) 125 to 150 bp; 3) 155 to 170 bp; and 4) 180 to 210 bp. These groups were defined to separate the microsatellite markers by size and thus avoid interference between markers within each multiplex. For each multiplex, compatibility of the primer pairs for each locus was tested with the software Multiplex Manager (1.2; [39]). As only four different colours of dye are available we set the maximum number of loci per reaction to four and a complementary threshold equal to seven (default parameter). Primer pairs were then successfully combined into six multiplexes, ranging from three to four markers per multiplex (Table 1).

**Table 1:** Summary statistics for the 22 microsatellite markers in *Microbotryum saponariae* (N = 97). Repeat: Motifs and repeat numbers of the microsatellite markers in the individual genome from which the markers were developed. Range: allelic size range in number of base pairs (bp). Multiplex: number indicating to which multiplex group the marker belongs. Multiplex 3 a and b were kept separate during PCR amplification to avoid the amplification interference that occurred when all four markers were amplified together, but they were pooled for genotyping in the end. Dye: forward primers of the selected markers were labelled with the following fluorescent dyes: Yakima Yellow equivalent to VIC, ATTO 550 equivalent to NED, ATTO 565 equivalent to PET. Alleles: number of alleles observed in the sample. H_O_: observed heterozygosity. H_E_: expected heterozygosity; Symbols (*) and (+) show significant deficit or excess in heterozygosity compared to Hardy-Weinberg expectations (* P < 0.05; ** P < 0.01; *** P < 0.001). F_IS_: Fixation index. SE: standard error.

Locus	Primer sequences (5'-3')	Repeat	Range	Multiplex	Dye	Alleles	Ho	He	FIS	
Msap_107	F: CAAGCTCCCCCACAGAGATG	(CA)_9_	110–119	1	FAM	2	0,20	0,46	0,55	***
	R: GAGACTCGCTCCGGACAAAA									
Msap_140	F: TGCGTTCACTCTTGCCTGAT	(GC)_8_	124–126	1	Yakima Yellow	2	0,00	0,05	1,00	***
	R: GAGCCCTAGGAACAGCGATC									
Msap_36	F: TCAAACCGAGATTCCGCCTG	(TG)_11_	168–176	1	ATTO 550	2	0,01	0,01	na	
	R: ATTGAGTTCGGTGCTGGGAG									
Msap_90	F: GTCGTATCCGGCTAGCTGAC	(TG)_10_	110–112	2	FAM	2	0,04	0,04	-0,01	
	R: AAAGTTGATCAGGGGCGTGT									
Msap_81	F: GTTCGCCACAAAGAGGGAGA	(CT)_10_	130–140	2	Yakima Yellow	4	0,04	0,06	0,32	*
	R: AGCGTTCAGAATGGCAAGGA									
Msap_11	F: GGATTTGACCAAGGCCGAGA	(TC)_8_	171–177	2	ATTO 550	3	0,00	0,08	1,00	***
	R: CTTGACGGTCGCCCAAAAAG									
Msap_4	F: TCCTTCTTCAGCTGCGGAAG	(GA)_13_	202–216	2	ATTO 565	4	0,14	0,50	0,71	***
	R: GGCGAGACACTACAAGAGGG									
Msap_94	F: CGGTGTCGGTCTCGTATGAG	(AGC)_11_	94–104	3b	FAM	3	0,17	0,26	0,38	**
	R: CGAGGATGAATCACGTCGGT									
Msap_73	F: AATGAGCATCGCGCCAAATC	(ACG)_10_	170–187	3a	ATTO 550	3	0,20	0,43	0,54	***
	R: CCAAAGAGGTTCCCGTGAGT									
Msap_139	F: CCATTCAATCTCGGAGGGGG	(GGT)_8_	214–217	3a	ATTO 565	2	0,00	0,04	1,00	***
	R: GACCGAAAGAGGCCACTAGG									
Msap_65	F: GTTCGAAGTTGAGGCGATGC	(GTC)_10_	103–108	4	FAM	3	0,05	0,17	0,69	***
	R: CCAGCACGGAGAGGAACTAC									
Msap_24	F: GCACAAGTCGTCGAACCCTA	(CCT)_8_	129–131	4	Yakima Yellow	2	0,00	0,04	1,00	***
	R: TCAGCGGTAGGGGTTAGTGA									
Msap_32	F: ACCGGAATCACGATCGACTG	(CGT)_9_	177–195	4	ATTO 550	3	0,01	0,05	0,80	***
	R: GCGAGAGGAGAGATTGAGCC									
Msap_96	F: CGATTCGCGACTACCTCCTC	(CTG)_9_	202–204	4	ATTO 565	2	0,05	0,45	0,88	***
	R: TCGCGACTCGGACATTGATT									
Msap_27	F: GTTTCGAAAGAGGGTGTGCG	(GGT)_11_	103–106	5	FAM	2	0,08	0,50	0,85	***
	R: GGTACTCTGCCCTCGACAAC									
Msap_77	F: CGAACGTTCCAGGGTCAGAA	(CCT)_8_	137–147	5	Yakima Yellow	2	0,02	0,06	0,66	***
	R: AAGAGAATGCGGATCAGCCC									
Msap_88	F: AGTACGCCGGTAAGATGGTC	(CCA)_8_	160–166	5	ATTO 550	2	0,00	0,02	1,00	**
	R: GTTAGGCTTCGAGTCCCTGG									
Msap_9	F: ATTCCCACTACAGTGCGAGC	(ACG)_9_	197–206	5	ATTO 565	2	0,00	0,02	1,00	**
	R: AATCTCCACCGCTCGATGTG									
Msap_146	F: AAGGACAAGAAGGCTGCCAA	(CGA)_9_	111–117	6	FAM	2	0,97	0,50	-0,95	***
	R: GGTTGGCGTTCATCATGTCG									
Msap_31	F: TAAAGACGCGTCCGACCATC	(TCG)_9_	129–132	6	Yakima Yellow	2	0,03	0,05	0,49	*
	R: GCGATCGCGGTCGTTTAATT									
Msap_102	F:CTCGCCCTTCCTTATAGCGT	(GCA)_14_	156–201	6	ATTO 550	11	0,40	0,58	0,32	***
	R:GTCCATGACGAAGGAGGTGG									
Msap_109	F:CGAGAGTGTCAAGGTGGTCC	(CGA)_11_	194–214	6	ATTO 565	3	0,08	0,21	0,64	***
	R:TGGCATCACCACCATCATCC									
**all**						51	0,11	0,21	0,61	
**SE**						0,465	0,046	0,044	0,100	

### Polymorphism of the Markers in Natural Populations

We further tested the polymorphism of the 22 microsatellite markers retained after the first screening described above, using 97 fungal individuals collected from 92 populations of *Sa*. *officinalis* and 5 populations of *Sa*. *ocymoides* across Europe (Fig 1, S2 Table). In addition, 24 individuals of a population of *M*. *saponariae* (population 1188, in Bures-sur-Yvette near the University Paris-Sud Campus, S2 Table) were also genotyped in order to assess within-population deviation from Hardy-Weinberg equilibrium. Diploid teliospores were harvested from one anther of a diseased sporulating flower. Fungal DNA was extracted in 100 μL using Chelex (Bio-Rad^®^, USA) protocol previously described [40], and ten-fold diluted for PCR amplification. Fungal samples were genotyped using the 6 multiplexes of microsatellite markers developed above. PCR reactions were performed separately for each multiplex in 12,5 μL volume containing 3 μL of DNA, ¼ of the recommended volume of multiplex PCR Kit (Qiagen^®^, USA), and 1.25 μL of the primer mix. The primer mix included 2 μM of unlabelled forward and reverse primer and 0.5 μM or 0.75 μM of labelled forward primer depending on the dye label. FAM and Yakima Yellow dyes were at 0.75 μM, while ATTO 550 and ATTO 565 dyes were at 0.5 uM. The PCR cycling program used was the same as described above. After checking for successful PCR amplifications on 2% (w/v) agarose gel electrophoresis, PCR amplicons were diluted and fractionated by capillary electrophoresis at the GENTYANE INRA platform as previously described. Alleles of each locus were scored using GENEMAPPER 5.0 (Applied Biosystems^TM^, USA).

### Cross-Species Amplification

To test the microsatellite markers developed for *M*. *saponariae* on other *Microbotryum* species, we sampled fungal individuals (S2 Table) and extracted DNA using the methods described for testing marker polymorphism in natural populations. The following species were tested: *M*. *lychnidis-dioicae* parasitizing *Si*. *latifolia*, *M*. *silenes-dioicae* parasitizing *Si*. *dioicae*, *M*. *silene-acaulis* parasitizing *Si*. *acaulis*, *M*. *lagerheimii* parasitizing *Si*. *vulgaris*, *M*. *violaceum s*.*s*. parasitizing *Si*. *nutans*, *M*. *violaceum s*.*l*. parasitizing *Si*. *caroliniana*, *M*. *violaceum s*.*l*. parasitizing *Si*. *flos-cuculi*, *M*. *shykoffianum* parasitizing *Dianthus pavonius*, *M*. *carthusianorum* parasitizing *D*. *superbus*, *M*. *dianthorum* parasitizing *D*. *seguieri* and *M*. *scabiosae* parasitizing *Knautia arvensis* (S2 Table). The success of amplification was checked on 2% (w/v) agarose gel electrophoresis and PCR amplicons were submitted to capillary electrophoresis at GENTYANE platform. For each locus, alleles were scored as previously described.

### Descriptive Statistics

The observed and expected heterozygosity (H_O_ and H_E_), departure from Hardy-Weinberg expectations (F_IS_), linkage disequilibrium (LD) among locus pairs and genotypic disequilibrium were calculated using GENEPOP [41] on the web at http://genepop.curtin.edu.au using the sample of one individual per population. Furthermore, F_IS_ was calculated within the population of *M*. *saponariae* for which all individuals were genotyped. The nominal P-value of 0.05 was adjusted for multiple comparisons using a Bonferroni correction (*i*.*e*., 2.3x10^-4^ based on 219 tests).

## Results and Discussion

The search for microsatellite loci in the *M*. *saponariae* draft genome yielded 188 loci meeting our criteria. We chose 96 loci located on different scaffolds and with different amplification sizes for designing multiplexes, with half of di- and half of tri-nucleotide repeats. Out of the 96 tested markers, four yielded no amplification. We thus screened six multiplexes of three to four microsatellite markers (Table 1, S1 Table) for polymorphism based on pooled DNA of a sample subset. We selected among those showing the highest level of polymorphism and the most easily readable peaks on the capillary electrophoresis chromatograms (S1 Fig).

We further tested the polymorphism of the markers retained after the first screening described above. A total of 22 microsatellite markers were found polymorphic and easy to score (Table 1). The number of alleles per locus varied from 2 to 11, for a total of 51 alleles observed. Descriptive statistics on the polymorphism of *M*. *saponariae* and on deviations from Hardy-Weinberg equilibrium are shown in Table 1. The mean observed (H_O_) and mean expected heterozygosity (H_E_) values ranged from 0 to 0.97 and from 0.01 to 0.58, respectively. Most markers (20 out of 22, *i*.*e*., 91%) displayed significantly lower levels of heterozygosity than expected under Hardy-Weinberg equilibrium. The fixation index (F_IS_) ranged from -0.95 to 1 for a mean multilocus value of 0.61 (±0.10). *Microbotryum saponariae* exhibited only 11% of heterozygous genotypes while 21% were expected under Hardy-Weinberg equilibrium, which suggests high selfing rates as previously reported in other *Microbotryum* species [16,40,42]. Only one marker, out of the 22, Msap_146, displayed higher levels of heterozygosity than expected (extreme FIS value of -0.95) with most individuals being heterozygous. Further analysis on the marker position in the *M*. *saponariae* genome revealed that it is located on the contig carrying the pheromone receptor gene. Most likely this marker is situated in the non-recombining region of the mating-type chromosome, which leads to permanent heterozygosity and high differentiation between the two mating types [25,29]. This marker will be useful for studies on mating types and mating system, but should not be used in analyses assuming Hardy-Weinberg equilibrium. Deficits in heterozygotes may also results from a Wahlund effect, which would be expected because we genotyped here only one individual per population and populations were sampled at a large geographical scale. If allele frequencies vary among populations, our pooled sample would necessarily have a deficit of heterozygotes [43]. In fact, the genotyping of a whole population showed little deviation from Hardy-Weinberg expectations (mean F_IS_ across loci, excluding SAP_146, of -0.03). On the other hand, despite a likely Wahlund effect, the heterozygote deficit observed here was lower than those previously reported within populations of *M*. *lychnidis-dioicae* (*e*.*g*., mean 0.96±0.06 in *M*. *lychnidis-dioicae*; [19]), suggesting lower selfing rates in *M*. *saponariae* than in *M*. *lychnidis-dioicae*. This is consistent with *M*. *lychnidis-dioicae* being bipolar and *M*. *saponariae* tetrapolar [44]. Bipolarity is indeed thought to evolve under selfing mating systems while tetrapolarity is beneficial under outcrossing, increasing discrimination against self [44].

After Bonferroni correction, significant linkage disequilibrium was observed only between six pairs of markers out of the 219 comparisons: Msap_11 and Msap_94, Msap_11 and Msap_31, Msap_4 and Msap_96, Msap_4 and Msap_27, Msap_96 and Msap_27, Msap_90 and Msap_77. This linkage disequilibrium may be due to genetic linkage between these markers or to population structure. Indeed, we pooled individuals from multiple populations and differences in alleles frequencies between populations will lead to apparent linkage disequilibrium when analysed as a single population.

Cross-species amplification was further examined among nine other *Microbotryum* species and was successful at 6 to 19 loci (Table 2). Some markers were amplified in all species, as for instance Msap_146 and Msap_73, while others, *e*.*g*. Msap_11 and Msap_4, were only amplified in the species most closely related phylogenetically to *M*. *saponariae*, such as the *Microbotryum* species on *Dianthus* hosts [3]. In general, the size of the alleles of each marker was different in the other species, except for nine markers where the alleles had identical size compared to those identified in *M*. *saponariae*. In some species, such as *M*. *scabiosae*, few markers could be amplified, unsurprisingly, given the high genetic distance from *M*. *saponariae* species [8]. Microsatellite markers in fungi are known to be difficult to amplify in other species, even closely related ones [45]. Among *Microbotryum* species, the divergence between the sister species *M*. *lychnidis-dioicae* and *M*. *silenes-dioicae* has been estimated to be 420,000 years old [46]. All the other species pairs studied here had older divergence times, as shown by phylogenies [3,7,30,46,47].

**Table 2:** Results of cross-species amplification of the 22 microsatellite loci in nine additional *Microbotryum* species. No amplification (-); successful amplification is given by the allelic profile of homozygous (colour scale) or heterozygous individuals (white). The name *M*. *violaceum s*.*l*. refers to the species complex name and it is indicated when the fungal species has not yet been described. The colour scale illustrates the allele size: darker colour corresponds to larger amplification products.

	Multiplex	1	1	1	2	2	2	2	
Host plant	Pathogen Locus	Msap_107	Msap_140	Msap_36	Msap_90	Msap_81	Msap_11	Msap_4	
*Silene latifolia*	*M*. *lychnidis-dioicae*	-	114/114	-	99/99	128/128	-	-	
*Silene dioicae*	*M*. *silene-dioicae*	-	114/114	142/142	99/99	128/128	-	-	
*Silene acaulis*	*M*. *silene-acaulis*	100/100	122/122	160/160	100/100	128/128	-	-	
*Silene vulgaris*	*M*. *lagerheimii*	-	124/124	-	108/108	-	-	-	
*Silene nutans*	*M*. *violaceum s*.*s*.	-	116/116	157/157	-	126/126	-	-	
*Silene caroliniana*	*M*. *violaceum s*.*l*.	-	122/122	-	95/95	-	-	-	
*Silene flos-cuculi*	*M*. *violaceum s*.*l*.	87/87	114/114	159/159	100/100	132/132	-	-	
*Dianthus pavonius*	*M*. *shykoffianum*	-	122/122	157/157	110/110	-	166/166	189/189	
*Dianthus superbus*	*M*. *carthusianorum*	-	122/122	157/159	110/110	-	166/166	190/190	
*Dianthus seguieri*	*M*. *dianthorum*	-	122/122	157/157	112/116	-	170/170	189/189	
*Knautia arvensis*	*M*. *scabiosae*	-	-	-	-	-	-	-	
	**Multiplex**	**3**	**3**	**3**	**4**	**4**	**4**	**4**	
**Host plant**	**Pathogen Locus**	**Msap_94**	**Msap_73**	**Msap_139**	**Msap_65**	**Msap_24**	**Msap_32**	**Msap_96**	
*Silene latifolia*	*M*. *lychnidis-dioicae*	93/93	166/166	219/219	72/72	122/122	166/166	-	
*Silene dioicae*	*M*. *silene-dioicae*	91/91	163/163	-	73/73	124/124	-	-	
*Silene acaulis*	*M*. *silene-acaulis*	81/81	160/160	-	85/85	124/124	175/175	160/160	
*Silene vulgaris*	*M*. *lagerheimii*	94/94	188/188	151/151	80/80	125/125	158/158	188/188	
*Silene nutans*	*M*. *violaceum s*.*s*.	87/87	189/189	174/174	80/80	122/122	166/166	-	
*Silene caroliniana*	*M*. *violaceum s*.*l*.	93/93	154/154	197/197	72/72	115/115	-	-	
*Silene flos-cuculi*	*M*. *violaceum s*.*l*.	93/93	166/166	198/198	85/85	128/128	-	-	
*Dianthus pavonius*	*M*. *shykoffianum*	82/87	185/185	-	-	-	-	-	
*Dianthus superbus*	*M*. *carthusianorum*	82/87	185/185	-	88/88	125/125	174/174	186/186	
*Dianthus seguieri*	*M*. *dianthorum*	82/87	183/183	-	88/88	115/124	183/186	186/186	
*Knautia arvensis*	*M*. *scabiosae*	-	154/154	-	-	110/110	-	-	
	**Multiplex**	**5**	**5**	**5**	**5**	**6**	**6**	**6**	**6**
**Host plant**	**Pathogen Locus**	**Msap_27**	**Msap_77**	**Msap_88**	**Msap_9**	**Msap_146**	**Msap_31**	**Msap_102**	**Msap_109**
*Silene latifolia*	*M*. *lychnidis-dioicae*	80/80	137/137	-	183/183	103/103	133/133	155/155	193/193
*Silene dioicae*	*M*. *silene-dioicae*	-	138/138	-	183/183	103/103	133/133	152/152	219/219
*Silene acaulis*	*M*. *silene-acaulis*	80/80	139/139	160/160	211/211	103/103	133/133	150/150	196/196
*Silene vulgaris*	*M*. *lagerheimii*	80/80	139/139	163/163	203/203	103/103	-	131/139	-
*Silene nutans*	*M*. *violaceum s*.*s*.	-	-	-	-	103/103	-	155/155	185/185
*Silene caroliniana*	*M*. *violaceum s*.*l*.	99/99	134/134	160/160	197/217	103/103	-	158/158	-
*Silene flos-cuculi*	*M*. *violaceum s*.*l*.	-	137/137	-	209/209	103/103	133/133	146/146	211/211
*Dianthus pavonius*	*M*. *shykoffianum*	100/100	134/140	174/174	209/209	108/108	-	158/158	191/191
*Dianthus superbus*	*M*. *carthusianorum*	83/83	137/137	174/174	209/209	108/108	-	158/164	-
*Dianthus seguieri*	*M*. *dianthorum*	90/97	137/143	174/174	209/209	108/108	-	158/158	97/203
*Knautia arvensis*	*M*. *scabiosae*	121/121	-	224/224	-	105/105	119/119	-	-

## Conclusion

The method used for screening polymorphism at microsatellite loci [35] was efficient and powerful. Using it enabled us to isolate 22 polymorphic markers grouped in 6 multiplexes without needing to develop the 96 specific fluorescent primers. The developed microsatellite markers will be very useful for future studies on the population structure and phylogeography of *M*. *saponariae* and for comparison with patterns in *M*. *lychnidis-dioicae* [18,40,48]. Furthermore, knowing the genome sequence and population structure of *M*. *saponariae* will allow the comparison of polymorphism at genes evolving under particular selection regime, such as the mating-type genes [49,50]. Microsatellites are also rapidly evolving markers that may reveal cryptic genetic subdivisions among populations parasitizing the two closely related host species *Sa*. *officinalis* and *Sa*. *ocymoides*, whose relationship remains unclear. It will also be interesting to assess whether *M*. *saponariae* is more outbreeding than the other anther-smut fungi studied so far [51]. Tetrapolar species are indeed usually outcrossing [44], and *M*. *saponariae* may reveal the first known tetrapolar selfing species [29].

The microsatellite markers will also be useful for studying the occurrence of multiple infections in *Sa*. *officinalis*, for assessing whether the results found in *Si*. *latifolia* [21,22] and in *Si*. *acaulis* [34] are general. In particular, we are interested in knowing whether the large long-lived *Sa*. *officinalis* plants carry multiple genotypes of *M*. *saponariae*, if they segregate spatially in different stems, and if genotypes co-occurring within the same host plant are significantly related.

In addition, the genomic resources released here will be useful for studies of fungal comparative genomics of anther-smut fungi and even more broadly. Fungi are indeed very useful models for tackling questions of genomic evolution and adaptation [52]. The published genome will help in particular studying mating-type chromosome evolution in the *Microbotryum* genus [25,26,29] and host specialization [1,30] in plant pathogenic fungi.

## Supporting Information

S1 Fig Capillary electrophoresis chromatograms with peaks of heterozygous individuals of *Microbotryum saponariae* at the polymorphic microsatellite loci 102 (Msap_102). Heterozygous individuals have the following allelic profile: a) 173 and 187; b) 173 and 196; c) 173 and 184.
(PDF)

S1 Table Template sequence used to design the primers for the 22 microsatellite markers in *Microbotryum saponariae*. Microsatellite repeats are highlighted in bold blue.
(PDF)

S2 Table Information on the *Microbotryum* individuals sampled from different host plants across Europe and analysed in this study. Individual fungal DNA was extracted using Chelex protocol (Biorad, USA) and was used to test the polymorphism of the 22 microsatellite markers in *M*. *saponariae* and in the other *Microbotryum* species used for cross-species amplification. Symbols (*) refers to DNA samples that were also extracted with the Nucleospin Soil kit (Macherey-Nagel, Germany) and were pooled as a template DNA for downstream applications in the first screening of markers polymorphism.
(PDF)
