# Supplementary figures and images for: Polymorphic Microsatellite Markers for the Tetrapolar Anther-Smut Fungus Microbotryum saponariae Based on Genome Sequencing
Source: PLoS One. 2016 Nov 10;11(11):e0165656. doi: 10.1371/journal.pone.0165656 (PMC5104459; doi:10.1371/journal.pone.0165656)

a)

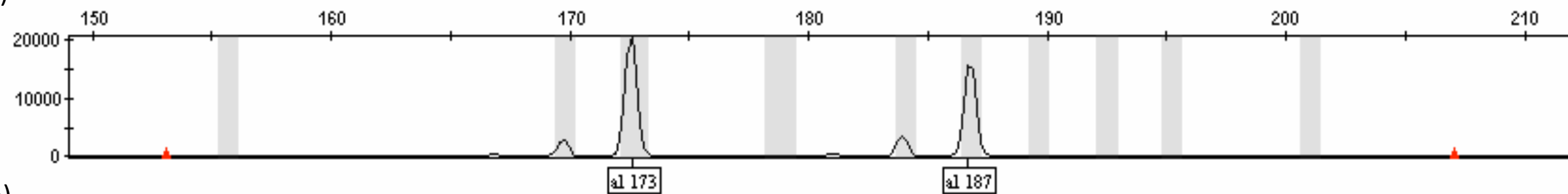

b)

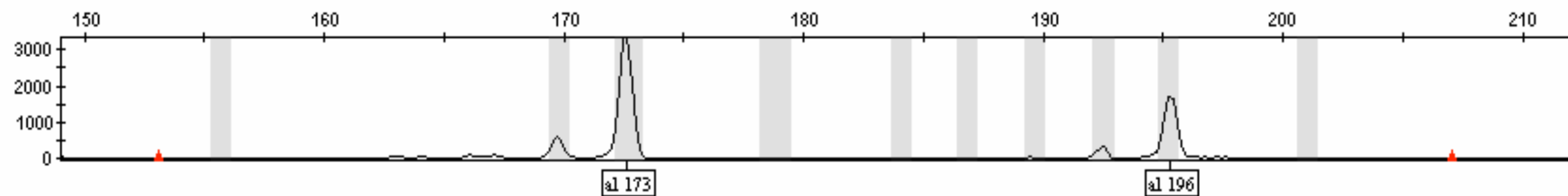

c)

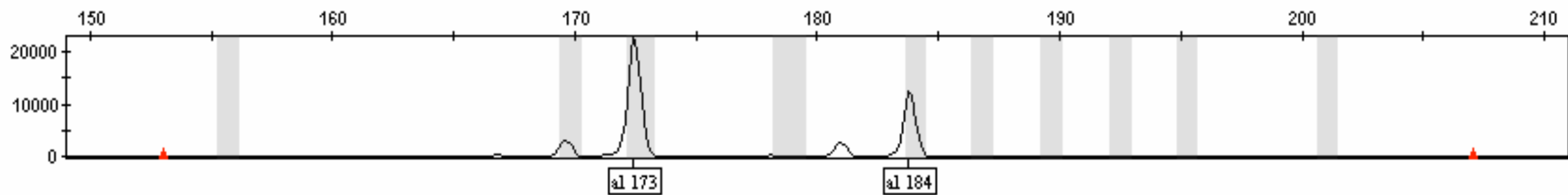

Supplement: S1 Fig — Heterozygous individuals have the following allelic profile: a) 173 and 187; b) 173 and 196; c) 173 and 184. (PDF) [file pone.0165656.s001.pdf]
