# Supplementary material for: Polymorphic Microsatellite Markers for the Tetrapolar Anther-Smut Fungus Microbotryum saponariae Based on Genome Sequencing
Source: PLoS One. 2016 Nov 10;11(11):e0165656. doi: 10.1371/journal.pone.0165656 (PMC5104459; doi:10.1371/journal.pone.0165656)
Supplement: S2 Table — Individual fungal DNA was extracted using Chelex protocol (Biorad, USA) and was used to test the polymorphism of the 22 microsatellite markers in M. saponariae and in the other Microbotryum species used for cross-species amplification. Symbols (*) refers to DNA samples that were also extracted with the Nucleospin Soil kit (Macherey-Nagel, Germany) and were pooled as a template DNA for downstream applications in the first screening of markers polymorphism. (PDF) [file pone.0165656.s003.pdf]

Strain	Pathogen	Host Plant	Sample origin	GPS coordinates		Sample Collectors
				Latitude	Longitude	
124*	*M. saponariae*	*Sa. officinalis*	France	42.883953	-0.024303	Tatiana Giraud; Michel Bartoli
137	*M. saponariae*	*Sa. officinalis*	France	45.289774	4.970755	Irène Till
341*	*M. saponariae*	*Sa. officinalis*	France	46.908638	4.723250	Jacqui Shykoff
408	*M. saponariae*	*Sa. officinalis*	France	45.216885	5.721551	Irène Till
420	*M. saponariae*	*Sa. officinalis*	Switzerland	46.306194	7.800417	Damien De Vienne; Jacqui Shykoff; Lucie Salvaudon
424	*M. saponariae*	*Sa. ocymoides*	Switzerland	46.407861	7.796667	Damien De Vienne; Jacqui Shykoff; Lucie Salvaudon
440	*M. saponariae*	*Sa. officinalis*	France	45.467846	5.541988	Damien De Vienne
441	*M. saponariae*	*Sa. officinalis*	France	42.886171	-0.023473	Michel Bartoli
559	*M. saponariae*	*Sa. officinalis*	France	44.972600	5.402136	Christian Raquin
561	*M. saponariae*	*Sa. officinalis*	Ukraine	51.053666	30.120182	Anders Møller
562*	*M. saponariae*	*Sa. officinalis*	Ukraine	51.06614	30.08732	Anders Møller
563	*M. saponariae*	*Sa. officinalis*	Ukraine	51.25471	29.57084	Anders Møller
570	*M. saponariae*	*Sa. officinalis*	Russia	55.788418	37.426501	Ilya Zakharov
571	*M. saponariae*	*Sa. officinalis*	France	42.530455	2.904619	Jean-Yves Hernet
572	*M. saponariae*	*Sa. officinalis*	France	43.764302	3.364393	Jean-Yves Hernet
591	*M. saponariae*	*Sa. officinalis*	France	44.139147	3.706936	Odile Jonot
721	*M. saponariae*	*Sa. officinalis*	France	46.975806	4.767479	Marc-André Selosse
722	*M. saponariae*	*Sa. officinalis*	Ukraine	51.277419	30.228838	Anders Møller
732	*M. saponariae*	*Sa. officinalis*	Germany	48.540167	9.048139	Kalman Vanky
827	*M. saponariae*	*Sa. officinalis*	Ukraine	49.961778	24.610611	Thierry Hamon
872	*M. saponariae*	*Sa. officinalis*	France	44.455964	3.929736	Pierre Gladieux
875	*M. saponariae*	*Sa. ocymoides*	France	43.780417	3.857083	Elisabeth Fournier
876	*M. saponariae*	*Sa. ocymoides*	France	44.651897	4.509450	Pierre Gladieux
892	*M. saponariae*	*Sa. officinalis*	France	43.998444	2.619333	Guislaine Refrégier
894	*M. saponariae*	*Sa. officinalis*	France	43.870309	3.118294	Guislaine Refrégier
895	*M. saponariae*	*Sa. officinalis*	France	43.015665	0.602661	Philippe Silar
896	*M. saponariae*	*Sa. officinalis*	Romania	45.794972	23.887083	François Delmotte
905	*M. saponariae*	*Sa. officinalis*	France	45.458750	4.768417	Marc-André Selosse
906*	*M. saponariae*	*Sa. officinalis*	France	45.159000	5.819694	Irène Till
913	*M. saponariae*	*Sa. ocymoides*	France	43.904600	5.297700	Pierre Gladieux
919	*M. saponariae*	*Sa. ocymoides*	France	43.843736	3.866389	Elisabeth Fournier
943	*M. saponariae*	*Sa. officinalis*	France	44.632516	4.253790	Pierre Gladieux
945	*M. saponariae*	*Sa. officinalis*	France	44.339956	0.530451	Pierre Gladieux
984	*M. saponariae*	*Sa. officinalis*	Ukraine	50.742500	29.572300	Kyrylo Savchenko
985	*M. saponariae*	*Sa. officinalis*	Ukraine	50.466300	30.544200	Kyrylo Savchenko
986	*M. saponariae*	*Sa. officinalis*	Ukraine	50.618100	30.460700	Kyrylo Savchenko
987	*M. saponariae*	*Sa. officinalis*	Ukraine	49.975900	31.348850	Kyrylo Savchenko
1047	*M. saponariae*	*Sa. officinalis*	France	44.597289	2.392086	Tatiana Giraud
1049*	*M. saponariae*	*Sa. officinalis*	France	45.143190	5.836523	Irène Till
1082*	*M. saponariae*	*Sa. officinalis*	France	48.699897	2.141674	Antoine Branca
1085	*M. saponariae*	*Sa. officinalis*	France	44.551114	1.772477	Tatiana Giraud
1178	*M. saponariae*	*Sa. officinalis*	Italy	44.238090	7.406950	Tatiana Giraud
1179	*M. saponariae*	*Sa. officinalis*	Italy	44.236370	7.406400	Tatiana Giraud
1184	*M. saponariae*	*Sa. officinalis*	Portugal	41.703249	-8.127364	Taiadjana Fortuna
1185	*M. saponariae*	*Sa. officinalis*	Portugal	41.703249	-8.129328	Antoine Branca
1188	*M. saponariae*	*Sa. officinalis*	France	48.698764	2.150616	Tatiana Giraud; Paola Bertolino
1190	*M. saponariae*	*Sa. officinalis*	France	48.700860	2.195613	Tatiana Giraud; Alodie Snirc; Stéphanie Le Prieur
1197	*M. saponariae*	*Sa. officinalis*	France	50.652698	3.187290	Huguette Robbe
1198	*M. saponariae*	*Sa. officinalis*	France	43.006605	-0.413666	Dany Roussel
1199	*M. saponariae*	*Sa. officinalis*	France	47.186208	3.005936	a botanist from the Tela Botanica network
1200	*M. saponariae*	*Sa. officinalis*	France	41.919608	8.903666	Nathalie Machon
1201	*M. saponariae*	*Sa. officinalis*	France	47.798531	3.333269	Micheline Boistard
1202	*M. saponariae*	*Sa. officinalis*	France	45.779764	15.018986	Matthias Lutz
1206*	*M. saponariae*	*Sa. officinalis*	Slovenia	48.467508	8.917728	Matthias Lutz
1207	*M. saponariae*	*Sa. officinalis*	Germany	48.533803	9.021781	Matthias Lutz
1208	*M. saponariae*	*Sa. officinalis*	Germany	48.458761	8.901319	Matthias Lutz
1214	*M. saponariae*	*Sa. officinalis*	France	45.149365	5.836866	Irène Till
1215	*M. saponariae*	*Sa. officinalis*	France	45.124356	3.823615	Elisabeth Civeyrac; Jean-Paul Civeyrac
1216	*M. saponariae*	*Sa. officinalis*	France	47.317974	0.405402	Emmanuelle Porcher
1218	*M. saponariae*	*Sa. officinalis*	France	43.736111	3.308889	Jean Lapeyrie
1224	*M. saponariae*	*Sa. officinalis*	Belgium	50.927864	4.326944	Fabienne van Rossum
1226	*M. saponariae*	*Sa. officinalis*	France	48.842813	2.361732	Manuela López-Villavicencio
1227	*M. saponariae*	*Sa. officinalis*	France	48.536255	2.261059	Didier Vigouroux
1228	*M. saponariae*	*Sa. officinalis*	France	48.695544	2.506675	Jean-Yves Dubuisson
1229	*M. saponariae*	*Sa. officinalis*	France	48.670509	2.407333	Thierry Derouin
1230	*M. saponariae*	*Sa. officinalis*	France	45.991422	3.132461	Gérard Guillot
1231	*M. saponariae*	*Sa. officinalis*	France	48.700694	2.123111	Gérard Leveslin
1232	*M. saponariae*	*Sa. officinalis*	France	50.428251	3.008348	Jean Walker; Anne-Sophie Walker
1234	*M. saponariae*	*Sa. officinalis*	France	50.341839	3.055517	Jean Walker; Anne-Sophie Walker
1235*	*M. saponariae*	*Sa. officinalis*	France	50.433649	2.852287	Jean Walker; Anne-Sophie Walker
1236	*M. saponariae*	*Sa. officinalis*	France	48.544583	2.162972	Gérard Leveslin
1240	*M. saponariae*	*Sa. officinalis*	Germany	51.443261	7.024945	Britta Büker
1241*	*M. saponariae*	*Sa. officinalis*	Germany	48.527228	9.018953	Matthias Lutz
1242	*M. saponariae*	*Sa. officinalis*	Italy	46.656306	10.878942	Matthias Lutz
1243	*M. saponariae*	*Sa. officinalis*	Austria	46.568492	13.869153	Matthias Lutz
1244	*M. saponariae*	*Sa. officinalis*	Austria	46.554072	13.907082	Matthias Lutz
1254*	*M. saponariae*	*Sa. officinalis*	Italy	44.319166	7.685159	Michael Hood
1255	*M. saponariae*	*Sa. officinalis*	Italy	44.328044	7.586110	Michael Hood
1256	*M. saponariae*	*Sa. officinalis*	Italy	44.332846	7.769385	Michael Hood
1257	*M. saponariae*	*Sa. officinalis*	Italy	44.373070	7.684244	Michael Hood
1258	*M. saponariae*	*Sa. officinalis*	Italy	44.397408	7.688976	Michael Hood
1286	*M. saponariae*	*Sa. officinalis*	France	48.701094	2.132835	Taiadjana Fortuna
1287	*M. saponariae*	*Sa. officinalis*	France	47.726278	2.593250	Richard chevalier
1288	*M. saponariae*	*Sa. officinalis*	France	49.001668	2.984552	Marion Eriksson
1289	*M. saponariae*	*Sa. officinalis*	France	47.411610	-0.440542	Francoise Menjon
1290	*M. saponariae*	*Sa. officinalis*	France	48.698833	2.150519	Bruno Lascaux
1291	*M. saponariae*	*Sa. officinalis*	France	50.483833	2.950028	JP Matysiak
1300	*M. saponariae*	*Sa. officinalis*	France	45.335917	2.757139	Manuela López-Villavicencio; Gennaro Coppa
1301	*M. saponariae*	*Sa. officinalis*	France	49.266818	0.354482	Manuela López-Villavicencio
1302	*M. saponariae*	*Sa. officinalis*	France	48.669215	2.486089	Simon Lang
1305	*M. saponariae*	*Sa. officinalis*	France	43.041722	0.379361	Jérome Enjalbert
1306*	*M. saponariae*	*Sa. officinalis*	Hungary	47.493694	19.097361	Levente Kiss
1307	*M. saponariae*	*Sa. officinalis*	Hungary	47.492740	19.354083	Levente Kiss
1308	*M. saponariae*	*Sa. officinalis*	France	43.171071	6.265580	Gilbert Gault
1309	*M. saponariae*	*Sa. officinalis*	France	45.814664	4.797119	Gilbert Gault
1310	*M. saponariae*	*Sa. officinalis*	France	45.861812	4.687377	Gilbert Gault
1311	*M. saponariae*	*Sa. officinalis*	France	45.800471	4.681711	Gilbert Gault
675	*M. lychnidis-dioicae*	*Silene latifolia*	Hungary	47.059167	19.406388	Gabor M. Kovacs; Timea Balazs
IT02 32-2 17A	*M. silene-dioicae*	*Silene dioicae*	Italy	46.047	9.623	Michael Hood
1248	*M. silene-acaulis*	*Silene acaulis*	France	45.027	6.275	Michael Hood
1253	*M. lagerheimii*	*Silene vulgaris*	France	45.4	6.11	Michael Hood
1249	*M. violaceum s.s.*	*Silene nutans*	Switzerland	46.777	10.16	Michael Hood
385	*M. violaceum s.l.*	*Silene caroliniana*	USA	36.911	-76.043	Michael Hood
1247	*M. violaceum s.l.*	*Silene flos-cuculi*	United Kingdom	55.78	-4.9	Michael Hood
1065	*M. shykoffianum*	*Dianthus pavonius*	Italy	44.189	7.688	Janis Antonovics
2-3	*M. carthusianorum*	*Dianthus superbus*	Italy	44.048	7.750	Michael Hood
1-3	*M. dianthorum*	*Dianthus seguieri*	Italy	44.482	7.281	Michael Hood
1118	*M. scabiosae*	*Knautia arvensis*	France	48.050	6.983	Dominik Begerow
